# Convergent evidence for a pitch deficit in hyperfunctional voice disorders

**DOI:** 10.1121/10.0037107

**Published:** 2025-07-07

**Authors:** Virginia Best, Turley Duque, Defne Abur, Cara E. Stepp

**Affiliations:** 1 Department of Speech, Language and Hearing Sciences, Boston University, Boston, Massachusetts 02215, USA; 2 Center for Language and Cognition Groningen, University of Groningen, Groningen, The Netherlands; 3 Department of Biomedical Engineering, Boston University, Boston, Massachusetts 02215, USAginbest@bu.edu, turleyd@bu.edu, d.abur@rug.nl, cstepp@bu.edu

## Abstract

A recent study in individuals with hyperfunctional voice disorders (HVDs) reported poorer *f*_o_ discrimination compared to matched controls. In that study, *f*_o_ discrimination was measured using each individual's own voice as the stimulus, which may have introduced confounds given the differences in vocal quality between the groups. Here, this paper addressed this possibility by using an *f*_o_ discrimination task where participants with and without HVDs judged the same external voice. In line with the previous study, the HVD group exhibited poorer *f*_o_ discrimination than controls, providing convergent evidence for a pitch-related auditory deficit in this population.

## 1. Introduction

Hyperfunctional voice disorders (HVDs) are the most frequently occurring type of voice disorder (Bhattacharyya, 2014) and account for up to 40% of referrals to multidisciplinary voice clinics (Roy, 2003). Traditionally HVDs have been associated with increased or imbalanced muscle tension (Hillman *et al.*, 2020), consistent with the “hyperfunctional” nomenclature, but the mechanisms behind this impaired voice production are not fully understood. Voice use (Altman *et al.*, 2005; Van Houtte *et al.*, 2011), psychosocial factors and personality (Roy *et al.*, 2000), and compensation for pre-existing pathologies (Hillman *et al.*, 2020) have all been implicated. There is also growing evidence suggesting auditory function as a contributing factor in HVDs (Abur *et al.*, 2021; Abur *et al.*, 2023; Nagy *et al.*, 2020; Nguyen *et al.*, 2022; Ramos *et al.*, 2018). Current models of vocal motor control, in which auditory feedback is a key component, illustrate how auditory deficits may result in persistent improper vocal control (Houde *et al.*, 2014; Weerathunge *et al.*, 2022). However, the empirical data supporting this idea are rather limited. In addition to its theoretical importance, a better understanding of the role of auditory acuity in HVDs could motivate new treatment approaches that target voice perception in addition to voice production.

The most compelling evidence to date for a role for auditory acuity in HVDs comes from a recent study by Abur *et al.* (2021). They examined the ability to use auditory feedback to update feedforward voice control in a large population of individuals with HVDs and reported a greater frequency of atypical responses relative to control participants. They also assessed fundamental frequency (*f*_o_) discrimination, using each individual's own voice as the stimulus, and found that participants in the HVD group needed larger differences in *f*_o_ to reliably notice a difference between two vowels (i.e., they had worse acuity) than controls. Although the use of own-voice stimuli ensured that the auditory task was directly related to each participant's auditory feedback, it also introduced a confound given the overall differences in vocal quality between the groups. Thus, this methodological choice may have influenced the results and/or increased variability in the data. In the current study, we revisited this issue in a new large sample of participants, this time using the same external vowel stimulus for all participants. In doing so, we sought to provide convergent evidence for a pitch-related auditory deficit in individuals with HVDs. Following Abur *et al.* (2021), our recruitment and analysis approach considered whether or not participants were singers. Singers have an increased risk of developing HVDs due to high voice use (Roy *et al.*, 2005) and are also more likely to have better pitch sensitivity as a result of vocal training compared to non-singers (Watts *et al.*, 2005). Thus, it is important to account for the influence of singing experience on pitch discrimination to avoid this factor obscuring differences between groups with and without HVDs.

## 2. Methods

### 2.1 Participants

A total of 120 individuals participated. The HVD group consisted of 17 non-singers (5 male,^1^ 12 female; age 39 ± 13 years) and 43 singers (5 male, 38 female; age 26 ± 10 years). The control group consisted of 17 non-singers (5 male, 12 female; age 39 ± 15 years) and 43 singers (5 male, 38 female; age 23 ± 5 years). Participants were classified as singers based on self-report (with their singing experience spanning professional, semiprofessional, student and teaching capacities).

All individuals with HVDs were diagnosed by a laryngologist based on a comprehensive voice evaluation in collaboration with speech-language pathology at the Massachusetts General Hospital Voice Center. None of the recruited individuals with HVDs had a history of neurological disorders or other speech, language, and hearing disorders. Control participants reported no history of neurological, voice, speech, language, or hearing disorders.

All participants were screened to ensure that they had normal (≤25 dB hearing level) pure-tone thresholds at standard audiometric frequencies from 500 to 4000 Hz. Screening was done using a model 109 or model 119 audiometer (Beltone Electronics Corp., Chicago, IL).

### 2.2 Procedures

These data were collected as part of a larger study focused on ambulatory monitoring of voice production in individuals with and without HVDs. The pitch discrimination task was conducted in a sound-attenuated booth. Stimuli were presented via a Komplete Audio 6 soundcard (Native Instruments GmbH, Berlin, Germany) and processed with an Eventide Eclipse V4 Harmonizer (Eventide, Inc., Little Ferry, NJ) before delivery via Sennheiser HD 280 Pro headphones (Sennheiser, Wedemark, Germany).

**Fig. 1.**
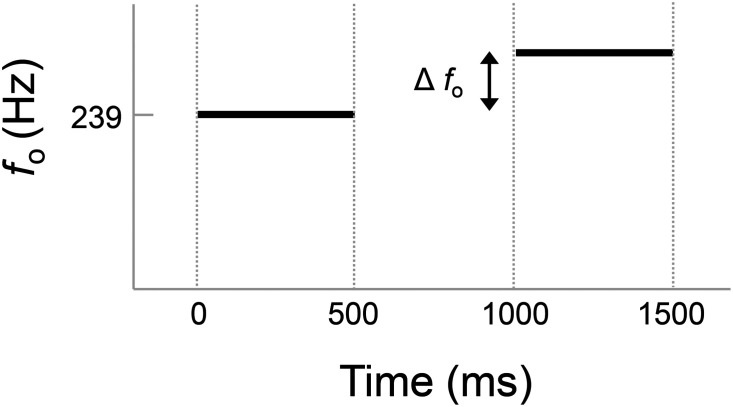
Schematic showing the stimulus design and trial structure.

Participants were presented with pairs of vowels and asked to judge whether the two stimuli sounded the “same” or “different” in terms of their pitch. The reference stimulus was a 500-ms segment of a steady vowel (/ɑ/) produced by a female talker with a mean *f*_o_ of 239 Hz. Prior to the start of the task, participants were played samples and asked to set the volume to a comfortable listening level; this level was not adjusted after the start of the task. Each trial consisted of one reference stimulus (the original recording) and one comparison stimulus with a shift in *f*_o_ (see Fig. 1). A cosine taper was applied to both stimuli such that the first and last 10 ms of each sample were equal to parts of a phase-shifted cosine. The order of the two stimuli was randomized, and the delay between them was 500 ms.

The *f*_o_ shift on the comparison stimulus was varied according to a standard adaptive procedure (García-Pérez, 1998), designed to find the *f*_o_ discrimination “threshold,” or the *f*_o_ shift at which a participant can reliably judge whether the stimuli are the same or different. Following Abur *et al.* (2021), the initial shift was +60 cents, with a 4-cent change following two correct responses (decreasing) or one incorrect response (increasing). The adaptive track was terminated after ten reversals or 60 trials, whichever occurred first. Thresholds were estimated by calculating the average *f*_o_ shift values in cents across the last four reversals, where the adaptive track had stabilized. Intermixed with the adaptive staircase were “catch trials” in which the reference stimulus was played twice to ensure attention to the task. Catch trials made up 20% of all trials, and responses to them were analyzed at the completion of the adaptive track. Catch trial accuracy was high in both groups (averages of 94% and 95% in the control and HVD groups, respectively).

## 3. Results

**Fig. 2.**
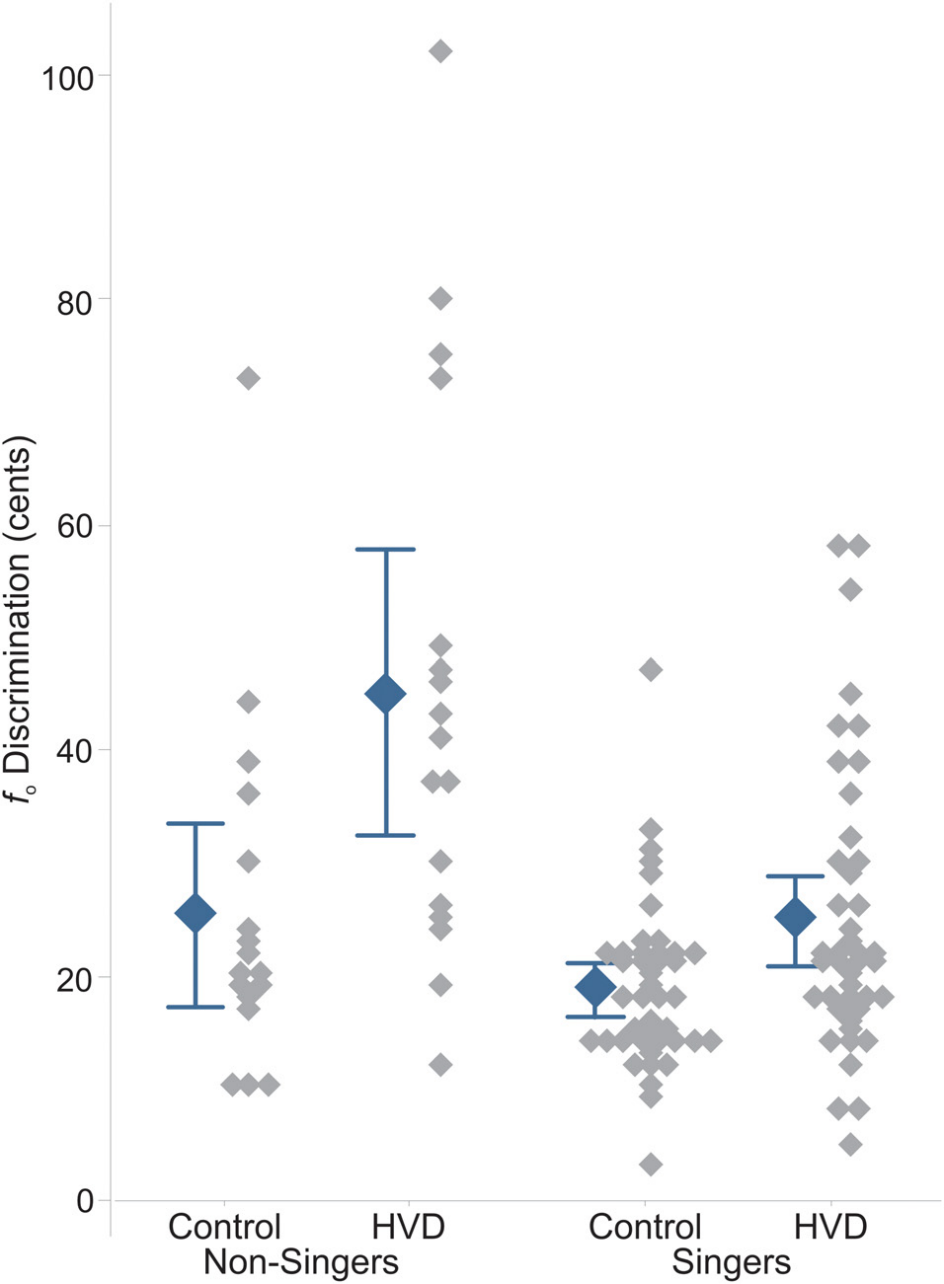
Individual and mean fundamental frequency (*f*_o_) discrimination thresholds (in cents) for non-singers (left) and singers (right) for the group with hyperfunctional voice disorders (HVDs) and the control group. Error bars show 95% confidence intervals.

Figure 2 shows individual and mean discrimination thresholds for the two groups (controls and HVD), separately for non-singers and singers. The first point to note is that there is substantial individual variability within each group and substantial overlap in the distributions across the four groups. A two-way analysis of covariance was conducted with a dependent variable of *f*_o_ discrimination threshold, independent variables of group (control, HVD) and singer status (non-singers, singers) as well as their interaction, and a covariate of age. This analysis revealed significant effects of group [*F*(1,115) = 20.49, *p* < 0.001, η^2^ = 0.15], with the HVD group having poorer thresholds than controls, and singer status [*F*(1,115) = 17.33, *p* < 0.001, η^2^ = 0.13], with non-singers having poorer thresholds than singers. There was also a significant interaction [*F*(1, 115) = 5.42, *p* = 0.022, η^2^ = 0.045], with the non-singers showing a larger mean difference between control and HVD groups than the singers. Age did not have a significant relationship with thresholds (*p* > 0.05).

The significant interaction between group and singer status reflects a difference in the magnitude of the group effect, which is also apparent in Fig. 2. In non-singers, the average *f*_o_ discrimination thresholds were 25.5 cents [standard deviation (SD) = 15.7 cents] in controls and 45.1 cents (SD = 24.4 cents) in speakers with HVDs, for an overall group effect of 19.6 cents. In singers, the average *f*_o_ discrimination thresholds were 18.9 cents (SD = 7.5 cents) in controls and 25.0 cents (SD = 12.8 cents) in speakers with HVDs, for an overall group effect of 6.1 cents. However, follow-up Student's *t* tests with a Bonferroni adjustment found significant differences between control and HVD groups for both non-singers [*T*(58) = 2.70, adjusted *p* value (*p*_adj_) = 0.009, *d* = 0.59] and singers [*T*(58) = 2.67, *p*_adj_ = 0.01, *d* = 0.98]. These analyses reveal that, despite the larger absolute difference found in non-singers, the effect size was in fact *smaller* in non-singers than in singers, attributable to the much higher variability in non-singers.

## 4. Discussion

The purpose of the current study was to compare *f*_o_ discrimination for an external voice stimulus in listeners with and without HVDs. The results revealed a robust *f*_o_ discrimination deficit in individuals with HVDs, in line with the findings of Abur *et al.* (2021). The current study provides convergent evidence for a pitch-related deficit in individuals with HVDs. An important modification made to the task in the current study was to employ the same external vowel stimulus for all participants, instead of using each participant's own production, to remove a potential source of bias and minimize stimulus-related variability. Discrimination thresholds were broadly consistent with those reported in Abur *et al.* (2021). In our control group, average values were 19 cents for singers and 26 cents for non-singers. These values are slightly lower than in the previous study (25 and 43 cents for singers and non-singers, respectively). In our HVD group, average thresholds were 25 cents for singers and 45 cents for non-singers. These values are again slightly lower than in the previous study (31 and 60 cents for singers and non-singers, respectively). Lower thresholds in the current study may reflect both differences in the stimuli and different cohorts of participants. Regarding participants, for example, we note that inclusion criteria related to hearing thresholds were slightly stricter in the current study. SDs were generally smaller in the current study than in the previous study, which we attribute primarily to the use of a consistent stimulus across participants. These overall results align with those of Abur *et al.* (2021) in that singers had substantially better pitch perception than non-singers overall, yet within singer and non-singer groups, individuals with HVDs had worse acuity than their respective controls.

Together, the current results and those of Abur *et al.* (2021) provide striking evidence that HVDs are associated with deficits in auditory processing (specifically *f*_o_ discrimination) regardless of whether it is a self-produced or externally produced voice. These findings bolster a small number of other observations in the literature of pitch-related deficits in individuals with voice disorders (Nguyen *et al.*, 2022; Ramos *et al.*, 2018). Importantly, in our sample all participants passed a hearing screening, potentially ruling out peripheral hearing loss as an underlying cause (as postulated previously by Nagy *et al.*, 2020). However, due to this inclusion criterion, we were unable to compare differences in audiometric thresholds between HVD and control groups; additionally, the typical hearing screening did not include 8 kHz or “extended high frequencies” (>8 kHz), which have shown promise at identifying subclinical hearing loss (Lough and Plack, 2022). Thus, it is currently unclear if the auditory profile of individuals with HVDs included peripheral hearing loss. Likewise, we do not know if the deficit is specific to pitch discrimination or extends to other suprathreshold auditory abilities such as loudness discrimination, modulation detection, and gap detection. A broader assessment of peripheral and central auditory function in this population is needed to understand the nature and scope of any deficit [see, for example, Naunheim *et al.* (2019)]. A more detailed auditory profile would also be valuable for interpreting auditory feedback-based vocal control in HVDs in terms of current theoretical models (Parrell *et al.*, 2019).

Further investigations are also needed to link pitch discrimination abilities to specific speech motor control processes. In the study of Abur *et al.* (2021), participants also completed pitch perturbation tasks intended to assess sensorimotor vocal learning. The results demonstrated that impaired sensorimotor learning was more common in the HVD group and also that this impaired sensorimotor learning was associated with pitch discrimination thresholds. They concluded that both atypical sensorimotor vocal learning and impaired voice production in HVDs may have an underlying basis in auditory function. Understanding the specificity of the auditory deficits in HVDs is an important next step to developing and testing a theoretical model for why these deficits result in the core symptoms of increased vocal effort and poor voice quality.

## Data Availability

These data were collected at Mass General Brigham, which is not allowed to give access to data without the principal investigator for the human studies protocol first submitting a protocol amendment to request permission to share the data with a specific collaborator on a case-by-case basis. This policy is based on strict rules dealing with the protection of patient data and information. Anyone wishing to request access to the data used for this study must contact Sarah DeRosa (sederosa@partners.org), Program Coordinator for Research and Clinical Speech-Language Pathology, Center for Laryngeal Surgery and Voice Rehabilitation, Massachusetts General Hospital.

## Footnotes

^1^
Gender information was not collected.
